# Idiopathic Chylous Pericardial Effusion Due to Lymphatic Malformation of Mediastinum

**DOI:** 10.7759/cureus.45298

**Published:** 2023-09-15

**Authors:** Animesh Mishra, Pinak P Das, Debasish Goswami, Amit Malviya, Manuj K Saikia, Reuben L Kynta, Donboklang Lynser, Diganta Buragohain, Preethi Preethi, Shashank Shashank

**Affiliations:** 1 Cardiology, North Eastern Indira Gandhi Regional Institute of Health and Medical Sciences, Shillong, IND; 2 Cardiothoracic and Vascular Surgery, Apollo Excelcare Hospital, Guwahati, IND; 3 Cardiothoracic and Vascular Surgery, North Eastern Indira Gandhi Regional Institute of Health and Medical Sciences, Shillong, IND; 4 Radiodiagnosis, North Eastern Indira Gandhi Regional Institute of Health and Medical Sciences, Shillong, IND

**Keywords:** sclerotherapy, pericardiectomy, pericardiocentesis, lymphangiectasia, chylopericardium

## Abstract

Chylopericardium can be due to a variety of secondary causes like trauma, radiation, tumors, following cardiac surgery, etc., or may be idiopathic due to abnormal lymphatic system and mediastinal lymphangiectasia, which is a rare entity. Here, we present a case of a 34-year-old previously healthy male presenting with idiopathic chylopericardium. 2D echocardiography revealed massive pericardial effusion without features of cardiac tamponade. Following pericardiocentesis, a CT scan of the thorax and MR lymphangiogram were done to arrive at a diagnosis of idiopathic chylopericardium. In addition to medical management, surgical treatment included partial pericardiectomy and sclerotherapy of the mediastinal lymphatic sac. The patient had an uneventful post-operative period.

## Introduction

Chylous pericardial effusion may occur due to a variety of secondary causes which include trauma, post-cardiac surgery, radiation, or tumors [1]. Rarely, it can be idiopathic due to lymphatic malformation and mediastinal lymphangiectasia. Idiopathic chylopericardium can occur in any age group and affects both sexes equally with children and young adults comprising the majority of cases [2]. Here, we present a case of idiopathic chylopericardium presenting as massive pericardial effusion. The patient was finally treated surgically with an uneventful post-operative period.

## Case presentation

**Figure 1:**
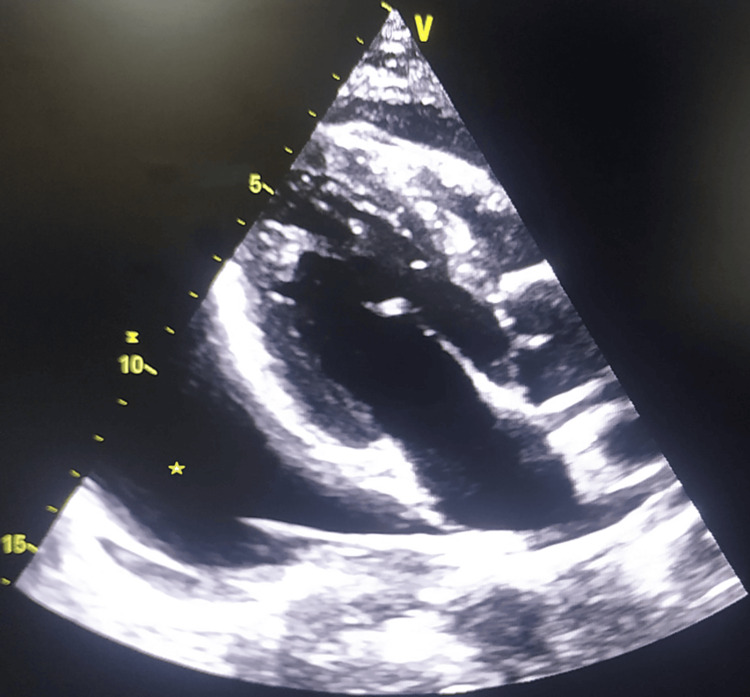
2D echocardiography image in parasternal long axis view showing massive pericardial effusion (asterisk)

A 34-year-old male patient presented to our hospital with chief complaints of easy fatiguability and shortness of breath for a period of three months. The patient also noticed a painless diffuse swelling on the right side of his neck for the same time period. There was no history of any significant past medical or surgical illness or any history of trauma. On examination, the patient’s vitals were stable. A soft non-tender swelling with an ill-defined margin was found in the right supraclavicular area. On evaluation, the chest x-ray was suggestive of an enlarged cardiac silhouette. The electrocardiograph was normal. A transthoracic 2D echocardiography was done to find out massive pericardial effusion without tamponade (Figure 1). Complete blood counts, liver, renal, and thyroid function tests, blood sugar, electrolytes, and lipid profiles were normal. Therapeutic pericardiocentesis was done and approximately 1.1 liters of milky pericardial fluid was drained out. A pigtail catheter was put which was in situ for a period of 14 days during which daily aspiration of pericardial fluid was done. Analysis of the fluid showed predominantly lymphocyte-rich fluid with few RBCs. Biochemical analysis revealed fluid protein 6.2 g/dL, sugar 98 mg/dL, triglycerides 1,145 mg/dL, and cholesterol 378 mg/dL. The cholesterol to triglyceride ratio was less than 1, which is suggestive of chylous fluid. Microbiological studies were negative, and no malignant cells were found on cytological analysis. Owing to these findings, a diagnosis of chylous pericardial effusion was made.

**Figure 2:**
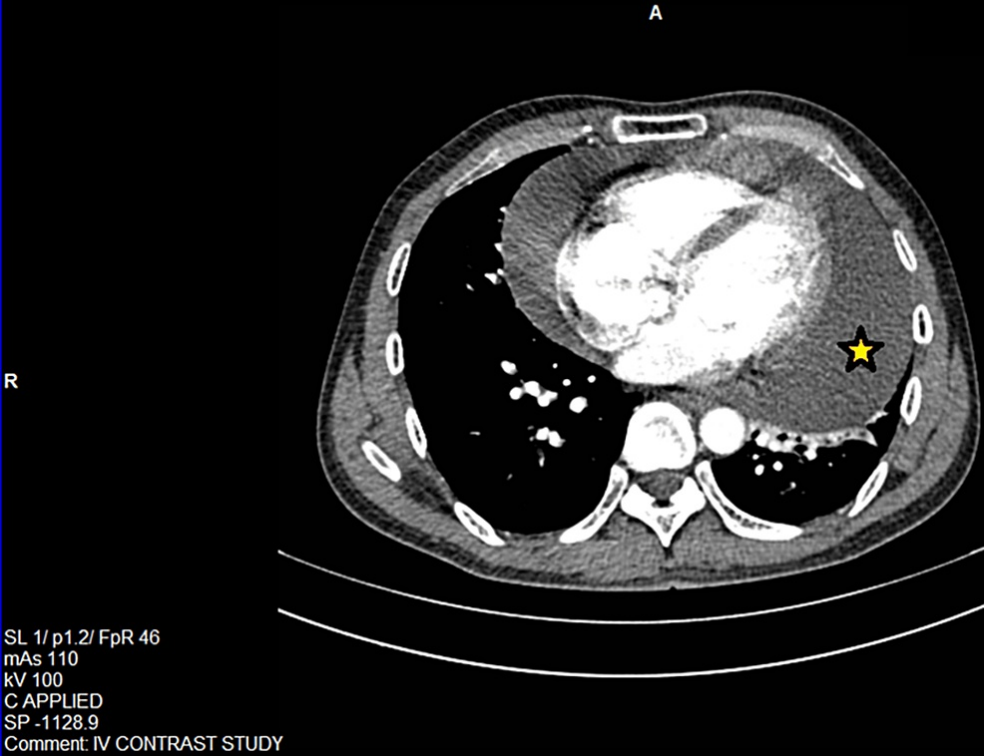
Axial contrast-enhanced CT in arterial phases at a mid-thoracic level in the mediastinal window showing normal size cardiac chambers with gross pericardial effusion (asterisk)

**Figure 3:**
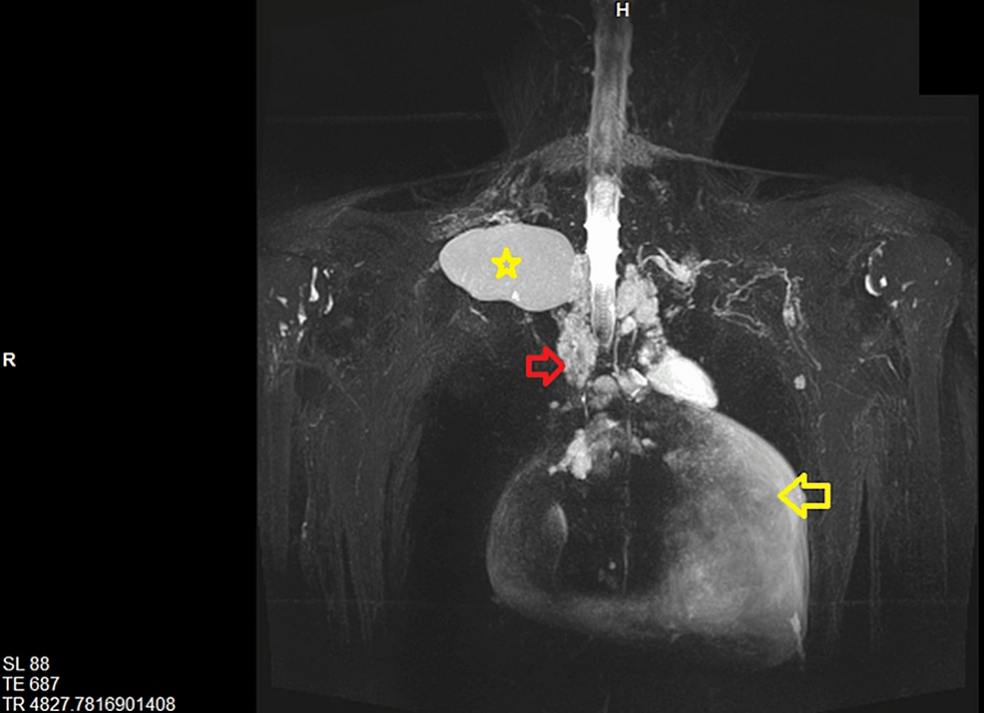
Coronal view of MR Lymphangiogram with 3D reconstruction showing well-defined T2 hyperintense collection in right supraclavicular location (asterisk) along with tortuous lymphatic mediastinal collaterals (red arrow) and gross pericardial effusion (yellow arrow)

A contrast CT scan of the neck and thorax was done which revealed multiple hypodense fluid attenuating lesions in bilateral supraclavicular regions, anterior and superior mediastinum along with pericardial effusion, suggestive of lymphatic malformation (Figure 2). To confirm our findings, an MR lymphangiogram of the thoracic duct, chest, and neck was done (Figure 3). A well-defined T2 hyperintense cystic lesion was seen at the right supraclavicular region along with multiple other cystic lesions in the left supraclavicular region, anterior mediastinum, and around the pericardium. In addition to that, gross pericardial effusion was seen without any definite communication with the other cystic channels. These findings altogether were consistent with lymphatic malformation.

The patient finally underwent surgery. Under general anesthesia, a partial pericardiectomy was done. Chylous pericardial fluid was drained with the excision of the retrosternal and extra-pericardial lymphatic sac. Additionally, aspiration and sclerotherapy of the right supraclavicular lymphatic sac were done with the installation of Bleomycin under general anesthesia. The patient had an uneventful pre- and post-operative period.

## Discussion

Chylopericardium, first described by Hasebrock in 1886, is a rare entity. Chylopericardium can be due to variety of causes like congenital mediastinal lymphangiectasia, iatrogenic after cardiac surgery, gastric signet ring cell carcinoma, Gorham syndrome (osteolysis that results from lymphangiomatosis with adjacent bone resorption), mediastinal malignant tumors like mediastinal dysgerminomas, blunt or penetrating trauma, infection, radiation, congenital anomalies of the lymphatic system, or primary idiopathic [1]. In 1954, Groves and Effler first reported the term primary isolated chylopericardium [3]. Since then, till November 2013, a total of 128 cases have been described in the literature [4]. Idiopathic chylopericardium refers to an abnormal lymphatic system and mediastinal lymphangiectasia causing chylopericardial effusions [5]. These abnormal lymphatic channels which may be a part of lymphangiomas or other lymphatic tumors, permit retrograde flow of lymph through them to the rich pericardial plexus causing primary chylous pericardial effusions [3].

Idiopathic chylopericardium can occur in any age group and affects males and females equally [2] with children and young adults comprising the majority of cases. Clinically, chylopericardium can have a wide spectrum of presentations, from asymptomatic that are incidentally diagnosed to cardiac tamponade. Chronic effusions that do not cause compression of the cardiac cavities may remain asymptomatic for a long time [3]. Cardiac tamponade ensues whenever there is cardiac compression and results in symptoms. As in our case, fatigue and exertional dyspnea are the most common symptoms [6]. Occasionally patients also complain of chest pain and palpitation. The patients who remain asymptomatic may have cardiomegaly on routine chest x-rays. Pericardial effusions can also be diagnosed on echocardiography, computerized tomography scan, or magnetic resonance imaging.

Pericardiocentesis is done to evaluate the characteristics of the pericardial fluid. Usually, whitish or milky chylous fluid is aspirated which has a high concentration of triglycerides and proteins [1]. Differential cell count reveals a predominance of lymphocytes [7]. Sudan III staining can be used to confirm the presence of fat globules. Blood cultures are usually negative [8].

Apart from CT scan and MRI, chylopericardium can be diagnosed non-invasively by imaging using Tc99m labeled red blood cells and oral administration of 131I-triolein [9]. These tests are used to establish the fistulous connections and to delineate the anatomy of the thoracic duct. They may also suggest a communication between the lymphatic system and the pericardial sac [9]. The literature review shows demonstrable abnormalities of thoracic lymphatic vessels in 80% patients who presented with cardiac tamponade and in 50% patients who developed tamponade after pericardiocentesis [10].

Once the diagnosis of chylopericardium is established and the cause is found to be idiopathic, the patient is started on treatment with a low-fat diet that is rich in medium-chain triglycerides. Other medical treatments include total parenteral nutrition and subcutaneous octreotide. Unlike in post-traumatic cases, idiopathic chylopericardium rarely responds to conservative treatment alone [9]. Surgical treatment becomes necessary and involves ligation and excision of the thoracic duct just above the diaphragm, along with partial pericardiectomy [7]. A pericardiectomy is performed to ensure complete drainage and to prevent later constrictive pericarditis. Thoracic duct ligation sometimes becomes an important part of the surgical procedure.

## Conclusions

To conclude, chylous pericardial effusion due to lymphatic malformation is relatively rare. The diagnostic approach mainly depends on echocardiography and analysis of the pericardial fluid with respect to cytological, biochemical and microbiological parameters. CT scan and MRI serve as necessary investigations to form the proper etiological diagnosis in these cases. Medical management is often unsuccessful in these cases. Therapeutic pericardiocentesis usually relieves symptoms of cardiac tamponade. Definitive surgical treatment which involves pericardiectomy, excision and sclerotherapy of other lymphatic sacs and thoracic duct ligation can be curative in most of the cases.
